# Biodegradation of high-molecular-weight polycyclic aromatic hydrocarbons by a novel species of the genus *Devosia* isolated from the deep-sea region of the Kermadec Trench

**DOI:** 10.3389/fmicb.2025.1584496

**Published:** 2025-07-14

**Authors:** Zefei Wang, Shanshan Zhao, Gen Chen, Shiwei Sun, Yue Liu, Haixin Chen, Liang Meng, Zhuang Han, Daoqiong Zheng

**Affiliations:** ^1^Hainan Institute, Zhejiang University, Sanya, China; ^2^Donghai Laboratory, Zhoushan, China; ^3^BGI Research, Sanya, China; ^4^Institute of Deep-sea Science and Engineering, Sanya, China

**Keywords:** polycyclic aromatic hydrocarbons, *Devosia polycyclovorans* Naph2^T^, pyrene, benzo[a]pyrene, whole-genome sequencing, transcriptome

## Abstract

Polycyclic aromatic hydrocarbons (PAHs) are categorized as persistent organic pollutants due to their high toxicity and environmental persistence. In this study, a deep-sea bacterium, designed Naph2^T^, was isolated from the sediments of the Kermadec Trench using PAH-enriched cultures. A comparative analysis of Overall Genome Relatedness Indices (OGRI) values between Naph2^T^ and closely related strains within the genus *Devosia* indicated that the isolate represents a novel species, designated as *Devosia polycyclovorans* sp. nov. (type strain Naph2^T^ = MCCC 1K09447^T^). This conclusion is further supported by physiological and biochemical analyses. Naph2^T^ exhibited the ability to degrade high-molecular-weight PAHs such as pyrene and benzo[a]pyrene, a feature not previously reported for any strain within the genus *Devosia*. The degradation degree of Naph2^T^ for pyrene and benzo[a]pyrene reached 58 and 48% at a concentration of 300 mg/L and 200 mg/L, respectively, in 5 days. Genomic analysis revealed key genes associated with PAH degradation, including aromatic ring-hydroxylating dioxygenase (RHD), *nagAa*, and downstream gene clusters such as *pht*, *pob*, and *pca*. Comparative genomic studies showed that Naph2^T^ harbors a greater number of PAH degradation genes than other species within the *Devosia* genus, demonstrate that it may have acquired these capabilities through horizontal gene transfer. Transcriptome data revealed significant upregulation of *pcaG* and *pcaH*, which encode enzymes involved in the degradation of 3,4-dihydroxybenzoic acid, a downstream intermediate of polycyclic aromatic hydrocarbon metabolism. These findings not only provide novel insights into the ecological roles of the genus *Devosia*, but also highlight the potential of this new species for PAH bioremediation applications.

## 1 Introduction

Polycyclic aromatic hydrocarbons (PAHs) are organic pollutants characterized by the presence of two or more benzene rings. High molecular weight polycyclic aromatic hydrocarbons (HMW-PAHs), consisting of four or more aromatic rings, demonstrate considerable environmental persistence. Their high molecular weight, chemical stability, and pronounced hydrophobicity contribute to environmental concentrations that often match or exceed those of low molecular weight PAHs (Xiang et al., 2018). Furthermore, HMW-PAHs are recognized for their high toxicity and carcinogenic potential (Suman et al., 2016), posing substantial risks to both ecosystems and human health (Sakshi and Haritash, 2020). Consequently, the development of effective strategies for the removal and degradation of these pollutants remains a critical focus within the field of environmental microbiology.

Remediation approaches for PAH contamination primarily encompass physical, chemical, and biological methods (Lors et al., 2012). Physical remediation techniques, such as adsorption and encapsulation, are straightforward but often incur high costs and may not achieve complete pollutant removal (Dai et al., 2022). Chemical remediation methods, such as chemical oxidation, are highly effective but can lead to secondary contamination and often require controlled environmental conditions (Kuppusamy et al., 2017). In contrast, bioremediation has garnered increasing scholarly attention due to its high efficiency, environmentally friendly, and sustainability. This process involves the microbial degradation of PAHs into simpler and less toxic compounds, thereby preventing secondary pollution and demonstrating adaptability to diverse environmental conditions, including extreme environments, which underscores its significant advantages (Haritash and Kaushik, 2009). The primary microorganisms responsible for the degradation of HMW-PAHs are predominantly Gram-positive bacteria, with *Mycobacterium* spp. being the most prevalent. Additionally, strains from the genera *Rhodococcus*, *Sphingomonas*, *Pseudomonas*, and *Cycloclasticus* are also involved (Huizenga et al., 2024; Song et al., 2021; Wang et al., 2018). Four *Mycobacterium* strains demonstrated the ability to use pyrene as their sole carbon source, achieving degradation rates of 48–75% for 100 mg/L pyrene within 7 days (Hennessee and Li, 2016). Furthermore, the PAH-degrading bacterium *Cycloclasticus* sp. P1, isolated from deep-sea sediments, achieved a pyrene degradation rate of 57% (Wang et al., 2018). Similarly, Lu screened and obtained microbial community from long-term PAHs-contaminated soil, and added this microbial community to the contaminated soil. After 35 days of incubation, the total amount of 16 optimally controlled PAHs in the contaminated soil was reduced from 95.23 mg kg^−1^ to 23.41 mg kg^−1^, while the total amount of PAHs in the control group with no degrading flora was reduced from 95.23 mg kg^−1^ to 72.73 mg kg^−1^ (Lu et al., 2019). The total amount of PAHs in the control group without degrading bacteria decreased from 95.23 mg kg^−1^ to 72.73 mg kg^−1^, which shows that microorganisms have good ability in remediation of PAHs contaminated environments (Lu et al., 2019).

To understand the degradation mechanisms and metabolic pathways of high-molecular-weight polycyclic aromatic hydrocarbons (HMW-PAHs), researchers have extensively utilized genomics, transcriptomics, and metabolomics. For example, studies on *Klebsiella michiganensis* EF4 and *Klebsiella oxytoca* ETN19, isolated from PAH-contaminated soil, employed genome sequencing and transcriptomic analysis to elucidate degradation pathways of phenanthrene and pyrene. These studies identified key genes including JYK05_14550, a novel gene proposed to initiate phenanthrene and pyrene degradation, and *yhfP*, encoding a putative quinone oxidoreductase. Additionally, genes encoding catechol 1,2-dioxygenase and quinone oxidoreductase were significantly expressed during PAH degradation (Lou et al., 2023). Although the analysis of multi-omics data has significantly contributed to elucidating the PAH degradation pathway, there is currently a lack of identified functional genes specifically encoding the degradation of HMW-PAHs. The PAHs ring hydroxylation dioxygenase (RHD) is a multicomponent degradative enzyme produced by bacteria, comprising two or three components, including the terminal oxygenase (large (*α*) and small (*β*) subunits) and the electron transport chain (Kweon et al., 2010). The degradation of PAHs is initiated by the hydroxylation of the benzene ring, a process catalyzed by RHDs, marking the first step in the biodegradation of PAHs. This hydroxylation, facilitated by cyclohydroxylated dioxygenase, is considered the most critical step in the entire PAH degradation pathway (Dionisi et al., 2004; Jakoncic et al., 2007). Within the *Mycobacterium* genus, the *nidAB* gene cluster, which encodes RHD, has been identified as one of the few gene clusters capable of degrading HMW-PAHs (Kweon et al., 2010). Specifically, during the degradation of pyrene by *Mycobacterium* sp. strain PYR-1, the production of 1,2-dihydrodiolpyrene and 4,5-dihydrodiolpyrene occurs at different initial sites of cyclohydroxylated dioxygenase activity. In previous studies, it has been demonstrated that 1,2-dihydrodiolpyrene undergoes a series of enzymatic reactions, resulting in the formation of 1,2-dimethoxypyrene, a compound that cannot be further metabolized. In contrast, 4,5-dioxydiolpyrene is converted into 3,4-dihydroxyphenanthrene through the action of dehydrogenase, cyclo-cleavage enzyme, and decarboxylase, subsequently entering the phenanthrene phthalic acid degradation pathway until complete mineralization is achieved (Kim et al., 2005; Moody et al., 2001).

The abyssal environment, characterized by extreme conditions such as high pressure, low temperature, and limited nutrient availability, has driven the diversification of species and genes (Hoshino et al., 2020), resulting in microorganisms with distinctive metabolic functions (Kweon et al., 2010). The cultivation and proliferation of microorganisms with specific metabolic capabilities can be facilitated by introducing selective carbon sources or substrates into the culture medium, thereby enabling the accumulation of microbial species capable of efficiently degrading particular substrates. In this study, a novel species of the genus *Devosia*, designated Naph2^T^, was isolated from sediments collected in the Kermadec Trench using enrichment cultures with phenanthrene as the sole carbon source. The degradation rates of various PAHs by Naph2^T^ were evaluated using high-performance liquid chromatography (HPLC). Through genomics, transcriptomics, and comparative genomic analyses, the key genes and metabolic pathways involved in PAH degradation by Naph2^T^ were identified. This work not only addresses ecological gaps within the genus *Devosia*, but also introduces a novel species with potential applications in the bioremediation of marine PAH pollution.

## 2 Materials and methods

### 2.1 Isolation, cultivation, and maintenance of strains

Sediment samples were collected from the Kermadec Trench, New Zealand (coordinates: −177.1914° E, −31.7985° N, at a depth of −9,779 m). The samples were subjected to enrichment culturing with pyrene as the sole carbon and energy sources. Enrichment cultures were established using a defined enrichment medium containing 0.2 g/L MgSO_4_·7H_2_O, 0.5 g/L CaCl_2_, 0.2 g/L K_2_HPO_4_, 0.25 g/L KNO_3_, and 0.01 g/L FeSO_4_·7H_2_O. The medium was sterilized by autoclaving at 121°C for 15 min prior to use. Under sterile conditions, 5 g of sediment sample was added to 50 mL of enrichment medium in 100 mL Erlenmeyer flasks. Pyrene was each added at a final concentration of 100 mg/L. Cultures were incubated statically at ambient temperature, with manual shaking every 12 h to promote oxygenation. The enrichment medium was replaced with fresh medium every two weeks, and the enrichment process was continued for two months. Individual bacterial strains were then isolated through dilution plating and microfluidic droplet sorting, following the methodology outlined by Kintses et al. (2010). The isolated species were subsequently purified on 2216E solid medium, which comprised 20 g/L Agar, 5 g/L Peptone, 1 g/L Yeast Extract, 0.1 g/L Ferric Citrate, 19.45 g/L NaCl, 5.98 g/L MgCl_2_, 3.24 g/L Na_2_SO_4_, 1.8 g/L CaCl_2_, 0.55 g/L KCl, 0.16 g/L Na_2_CO_3_, 0.08 g/L KBr, 0.034 g/L SrCl_2_, 0.022 g/L H_3_BO_3_, 0.004 g/L Na_2_SiO_3_, 0.0024 g/L NaF, 0.0016 g/L NH_4_NO_3_, and 0.008 g/L Na_2_HPO_4_, with the final pH adjusted to 7.6 ± 0.2. The incubation was conducted at 30°C for 6 days. The isolates were obtained after several subculturing. These isolates were further characterized based on their color and morphology, and preserved at −80°C in suspensions containing 25% (v/v) glycerol.

### 2.2 Phenotypic, biochemical and chemotaxonomic characterization

Morphology was studied by scanning electron microscopy. The strain Naph2^T^ was cultured on 2216E solid medium for two days. A 1 cm^2^ section of the medium was excised and placed in an Eppendorf tube, where it was submerged in a 2.5% glutaraldehyde solution to preserve the bacterial cells. Subsequently, the samples were washed twice with phosphate buffer, followed by treatment with a graded ethanol series (ethanol concentrations of 50, 70, 90, and 100%). After processing through tert-butanol, all samples were freeze-dried by the lyophilizer. The dried samples were then mounted on conductive adhesive tape, sputter-coated with a gold layer, and examined using scanning electron microscopy (SEM). The growth temperature parameters were evaluated using 2216E liquid medium, which contains peptone and yeast extract as the primary energy and carbon sources, across a temperature range of 0–40°C (specifically at 0, 4, 10, 20, 25, 28, 30, 33, 37, and 40°C). For pH tolerance assessment, 2216E broth was prepared with pH values ranging from 4.0 to 10.0 in 0.5-unit intervals, pH was maintained during incubation using citrate, phosphate, and Tris–HCl buffers, and growth was monitored after a 2-day incubation period. Sodium chloride tolerance was determined using 2216E broth supplemented with sodium chloride concentrations ranging from 0 to 5% (w/v) in 0.5% (w/v) increments, incubated at 28°C for 2 days on a rotary shaker. The growth of the species Naph2^T^ was quantified using a UV spectrophotometer (UV-1800; Shimadzu) at 600 nm. The susceptibility of strain Naph2^T^ to common antibiotics was evaluated using the paper diffusion method, and the resistance profile was determined by measuring the diameter of the inhibition zone. The utilization of sole carbon and energy sources was tested following the methodology of Ye et al. (2019). The isolates underwent further biochemical characterization utilizing BioMérieux’s API ZYM and API CH systems, according to the manufacturer’s protocols. For fatty acid analysis, the cells were cultured on 2216E plates for 3 days at 30°C. Whole-cell fatty acids were extracted and analyzed in accordance with the guidelines of the Sherlock™ Microbial Identification System MIDI, employing the TSBA6 database (Version 6.0). Two closely related reference strains, *Devosia marina* L53-10-65^T^ and *Devosia subaequoris* HST3-14^T^, were acquired from the Marine Culture Collection of China (MCCC) (Liu et al., 2020) and the China General Microbiological Culture Collection Center (CGMCC) (Lee, 2007), respectively. They were cultured under the same conditions for comparative analysis.

### 2.3 16S rRNA gene sequencing and phylogenetic analysis

For 16S rRNA gene sequencing and phylogenetic analysis, the 16S rRNA gene was amplified via PCR using universal bacterial primers 27F (5′-AGAGTTTGATCCTGGCTCAG-3′) and 1492R (5′-GGTTACCTTGTTACGACTT-3′) with the PCR MasterMix from G-clone (Beijing) Biotech Co., Ltd., in a 20 μL PCR system. The PCR products were sequenced using the amplification primers at Zhejiang Shangya Biotechnology Co., Ltd. The 16S rRNA gene sequence of strain Naph2^T^ has been submitted to the GenBank database. Almost full-length 16S rRNA gene sequence of strain Naph2^T^ was obtained and aligned with corresponding sequences of type strains of the genus *Devosia* obtained from the EzBioCloud database.^1^ Multiple sequence alignments were performed using Muscle software with default parameters (Edgar, 2004), and these sequences were constructed using Gblocks (Chang et al., 2015). The phylogenetic tree, based on 16S rRNA gene sequences, was reconstructed using IQ-TREE software version 2.1.2. This reconstruction employed the maximum likelihood (ML) method under the TN + F + I + G4 nucleotide substitution model, as determined by ModelFinder (Kalyaanamoorthy et al., 2017; Minh et al., 2020). To evaluate the reliability of the tree topology, phylogenetic trees were constructed using the Bootstrap method with 1000 replicates.

### 2.4 Whole genome sequencing and analysis

The genomic DNA was extracted using the Takala MiniBEST Bacteria Genomic DNA Extraction Kit Ver.3.0. Illumina MiSeq and Nanopore sequencing platforms were used to sequence the whole genome of strain Naph2^T^, with all sequencing conducted at BGI. AdapterRemoval (Schubert et al., 2016) and SOAPec (Luo et al., 2012) were employed to eliminate joint contamination and filter low-quality reads, respectively. Subsequently, Flye software was utilized to assemble the data from the Nanopore sequencing platform to obtain the contig sequences (Kolmogorov et al., 2019). The high-quality second-generation data were then used to correct the third-generation contig results using Pilon (Walker et al., 2014). Furthermore, overall genome relatedness indices (OGRI), including digital DNA–DNA hybridization (dDDH) (Auch, n.d.) average nucleotide identity (ANI), and amino acid identity (AAI), were assessed. Whole genomes of *Devosia* species phylogenetically related to Naph2^T^ were obtained from the NCBI online database.^2^ The ANI, AAI, and dDDH values between strain Naph2^T^ and closely related strains were determined using FastANI (Version 1.33) (Jain et al., 2018) and CompareM,^3^ and Genome-to-Genome Distance Calculator (GGDC) version 3.0 (Meier-Kolthoff et al., 2022). The ANI, AAI and DDH values were visualized using the Heatmap tool of the software Chiplot.^4^ GeneMarkS and Prokka were employed for the prediction of protein-coding genes and the identification of open reading frames (ORFs) (Besemer, 2001; Seemann, 2014). Additional genetic elements were predicted using various tools: tRNAscan-SE for tRNA prediction (Chan et al., 2021), Barrnap software for rRNA prediction,^5^ IslandPath-DIOMB for the identification of genomic islands (GIs) (Bertelli and Brinkman, 2018), and CRISPRCasfinder for the detection of CRISPR elements (Couvin et al., 2018). Functional annotation of genes was conducted using DIAMOND software (Buchfink et al., 2021) to compare coding-protein gene sequences against the NR (Non-Redundant Protein Sequence Database), KEGG (Kyoto Encyclopedia of Genes and Genomes) and COG (Clusters of Orthologous Groups) databases. The annotation results from KEGG and NR were integrated to identify genes involved in the degradation of PAHs.

### 2.5 Degradation of pyrene and benzo[a]pyrene by individual Naph2^T^

Strain Naph2^T^ was cultured in 2216E liquid medium at 30°C with agitation at 180 rpm until reaching the logarithmic growth phase. The incubation was conducted using inorganic salt medium (MSM), which comprised 5 g/L NaCl, 1 g/L NH_4_SO_4_, 1 g/L K_2_HPO_4_, 1 g/L KH_2_PO_4_, 0.35 g/L MgSO_4_·7H_2_O, 0.05 g/L FeSO_4_, and 0.02 g/L yeast extract, with the pH adjusted to 7.2. Pyrene and benzo[a]pyrene were dissolved in dimethyl sulfoxide and thoroughly mixed into the medium to achieve final concentrations of 100 mg/L, 200 mg/L and 300 mg/L for pyrene, and 50 mg/L, 100 mg/L and 200 mg/L for benzo[a]pyrene, respectively. The degradation experiments were carried out in 100 mL conical flasks. Pre-cultivated strains were adjusted to an optical density at 600 nm (OD600) of 1.0 and inoculated into 30 mL of MSM medium at an inoculum volume of 10%. An inorganic salt medium containing only the PAHs substrate, with no bacterial inoculation, was used as a negative control. At 24-h intervals, the culture system at the corresponding time point was taken out from a thermostatic shaker, to which 30 mL of hexane was added with sufficient vigorous shaking to promote the efficient extraction of PAHs from the aqueous phase into the hexane. The upper hexane layer was carefully collected and transferred to a rotary evaporation vial. After concentration via rotary evaporation, the residue was redissolved in 1.5 mL of methanol, filtered through a 0.22 μm polyethylene membrane filter, and subjected to HPLC analysis. The HMW PAHs concentration changes were quantitated by Agilent 1,260 Infinity II HPLC with a TC-C18 reversed-phase column (Agilent, 4.6 mm × 250 mm, 5 μm particle size) on a gradient elution system at a flow rate of 1.0 mL/min, and with detection wavelengths set at either 254 or 280 nm. The residual concentration of pyrene and benzo[a]pyrene in the incubation solution was determined based on a standard curve, and the degradation rate was calculated using the following formula: Degradation rate (%) = [(Initial concentration−Residual concentration)/Initial concentration] × 100%. Subsequently, the degradation curve was plotted using GraphPad Prism 10.

### 2.6 Comparative genomic analysis of the strains from genus *Devosia*

The dataset utilized for the pan-genomic analysis comprised Naph2^T^ and its 29 neighboring species. The Bacterial Pangenome Analysis (BPGA) pipeline, as described by Chaudhari et al. (2016), was employed to conduct the pangenome analysis using default parameters. The pangenome size of the genus *Devosia* was modeled using a power-law regression function, f(x) = a * x*^b^*, in which ‘f(x)’ represents the total number of gene families, ‘x’ denotes the number of genomes analyzed, and ‘b’ is a free parameter. If the index ‘b’ was less than zero, the pangenome of the genus Devosia was suggested to be “closed.” It suggesting that the pangenome size remains relatively constant despite the addition of new genomes. Conversely, if the value of ‘b’ lies between zero and one, the pangenome is considered “open.” The core genome size of the genus *Devosia* was fitted using an exponential decay function, f(x) = c * e^(dx)^, with a built-in program of the BPGA, in which ‘f(x)’ represents the number of core gene families, and ‘c’ and ‘d’ are free parameters. Orthologous genes were identified with the USEARCH algorithm using a threshold of 0.5. Since changes to the similarity threshold to 0.3, 0.4, 0.6, and 0.7 did not substantially change the number of gene families, 0.5 was selected as the default value. Pangenome/core genome plots were calculated over 500 iterations.

### 2.7 Transcriptomic sequencing and analysis

Strain Naph2^T^ was cultivated in two distinct media to investigate its growth and metabolic responses: a glycerol medium, consisting of minimal salts medium (MSM) supplemented with 1% glycerol, and a pyrene medium, comprising MSM supplemented with 200 mg/L pyrene, the one with glycerol added is the control group and the one with pyrene added is the experimental group. The cultures were maintained at 30°C with continuous shaking at 180 rpm for 4 days. Upon reaching the exponential growth phase, bacterial cells were collected via centrifugation at 4°C (5,000 × g for 10 min) to separate them from the culture medium. The resulting cell pellets were rapidly frozen in liquid nitrogen to facilitate RNA extraction. RNA was isolated using the RNAprep Pure Kit (Tiangen, China) in accordance with the manufacturer’s instructions. The quality and concentration of the extracted RNA were evaluated using a NanoDrop spectrophotometer and agarose gel electrophoresis. For RNA sequencing, total RNA was sequenced on the Illumina platform employing Next-Generation Sequencing (NGS) technology at Shanghai Meiji Biomedical Technology Co., Ltd. Prior to alignment, the raw reads underwent filtering to eliminate low-quality sequences and adaptors. The filtered sequences were aligned to the reference genome using Bowtie2 (Langmead and Salzberg, 2012). RSEM^6^ was employed for the quantitative analysis of gene expression, followed by the application of DESeq2 (Love et al., 2014) for the analysis of differentially expressed genes. Subsequently, KEGG and GO (Gene Ontology) pathway enrichment analyses were conducted on the differential genes.

## 3 Results

### 3.1 Morphological, physiological and biochemical characteristics of Naph2^T^

In this study, the strain Naph2^T^ was isolated from phenanthrene-enriched sediment samples. Scanning electron microscopy analysis demonstrated that the isolate Naph2^T^ exhibited a typical rod-like morphology (0.3–0.5 × 1–2 um) (Supplementary Figure S1), with rough surfaces and distinct folds and textural structures. The species Naph2^T^ demonstrated optimal growth at 30°C and pH 7.5, with a tolerance of up to 3.5% (w/v) NaCl, and was most effectively cultivated on 2216E medium. In comparison, *Devosia marina* L53-10-65ᵀ showed optimal growth at 28°C and pH 7.0–8.0, with a NaCl tolerance of up to 2% (w/v), while *Devosia subaequoris* HST3-14ᵀ was able to grow across a broader range of 20–42°C and pH 5.1–12.1. Notably, antibiotics such as penicillin and ampicillin significantly inhibited the growth of Naph2^T^, whereas closely related strains (*Devosia marina* L53-10-65^T^ and *Devosia subaequoris* HST3-14^T^) exhibited normal growth under the same antibiotic stress. Strain Naph2ᵀ was able to ferment D-glucose, D-mannitol, and N-acetyl-D-glucosamine, but was unable to utilize D-fructose and L-sorbitol. In contrast, the closely related strains *Devosia marina* L53-10-65ᵀ and *Devosia subaequoris* HST3-14ᵀ tested positive for D-fructose and L-sorbitol, indicating clear phenotypic differences in carbohydrate metabolism within the genus *Devosia*. In enzyme activity assays, Naph2^T^ exhibited positive activity for trypsin, whereas two comparable strains demonstrated negative reaction of the substance. Regarding the utilization of sole carbon sources, Naph2^T^ was capable of metabolizing glucose, in contrast to HST3-14^T^, which was not. Conversely, Naph2^T^ was unable to metabolize mannitol, while L53-10-65^T^ successfully utilized it. Furthermore, significant differences were observed in the major fatty acid composition among the three species. Naph2^T^ exhibited a low C_18:0_ content (4.71%), whereas HST3-14^T^ displayed a considerably higher content at 17.5%. Additionally, the C_18:1_ ω7c 11-methyl content exceeded 20% in both Naph2^T^ and L53-10-65^T^ strains, whereas this component was absent in HST3-14^T^. Further detailed physiological and biochemical properties are provided in Table 1. The findings from physiological and biochemical analyses indicated that Naph2^T^ shares substantial similarities in biological characteristics with the genus *Devosia*, distinguishing it from evolutionarily related strains and suggesting it may represent a novel species.

**Table 1:** Differential characteristics of strain Naph2^T^ and its closely related species.

Characteristic	1	2	3
Antibiotic sensitivity			
Penicillin (10 U/silce)	−	+	+
Ampicillin (10ug/silce)	−	+	+
Carbenicillin (100ug/silce)	−	+	+
Piperacillin (30ug/silce)	−	+	+
Cefamezim (30ug/silce)	−	+	+
Test of API 50CH			
D-Ribitol	+	−	−
D-Fructose	−	+	+
L-Sorbose	−	+	−
Inositol	−	+	+
N-Acetylglucosamine	−	+	−
D-Melibiose	−	+	+
D-Raffinose	−	+	+
Starch	+	−	−
Xylitol	−	+	−
Test of API ZYM			
Valine arylamidase	+	+	−
Trypsin	+	−	−
*β*-Glucuronidase	+	+	−
Sole carbon utilisation			
D-Glucose	+	+	−
Mannitol	−	+	−
lactic acid	+	−	w
Major fatty acid composition (%)			
C_16:0_	10.12	19.5	15.9
C_18:0_	4.71	6.3	17.5
C_18:1_	43.9	36.4	48.6
C_18:1_ ω7c 11-methyl	21.64	21.8	NA

1, Naph2^T^; 2, D. marina L53-10-65^T^; 3, D. subaequoris HST3-14^T^. +, Positive reaction; −, Negative reaction; w, Weakly-positive reaction; NA, None of data. All data were generated in this study.

### 3.2 Phylogenetic analysis based on 16S rRNA sequence

**Figure 1:**
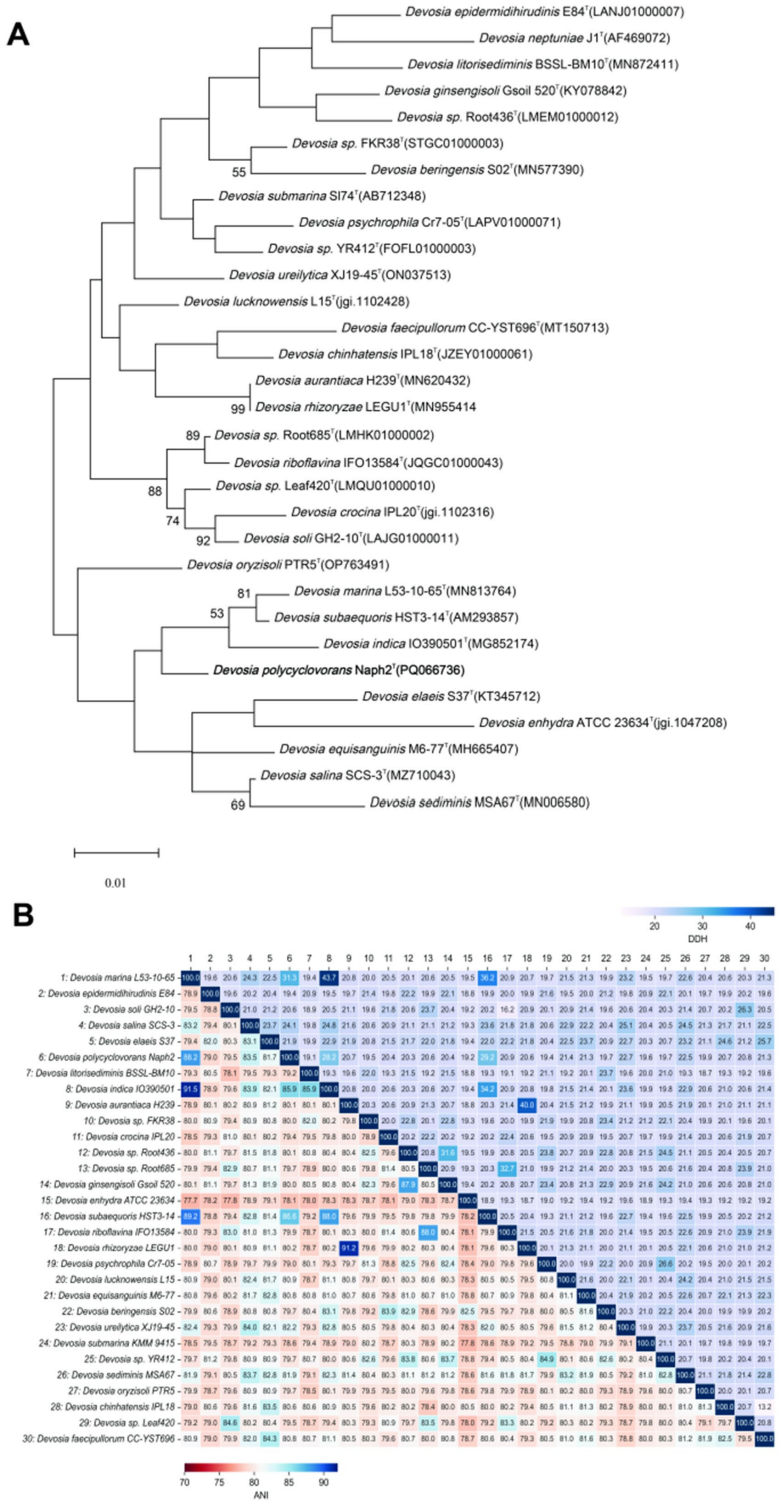
Phylogenetic relationship based on 16S rRNA and whole-genome analysis of the strain Naph2^T^ with other closely related species. **(A)** The phylogenetic analysis of the strain Naph2^T^ and its most closely related strains of the genus *Devosia* was conducted using the maximum likelihood method. Bootstrap values, derived from 1,000 replicates, are indicated by node colors corresponding to their respective values. Accession numbers in the phylogenetic tree are sourced from the ezBiocloud and NCBI database. Typical strains are denoted by the letter “T.” **(B)** A heatmap of ANI and DDH values among Naph2^T^ and related taxa species was utilized to evaluate the taxonomic status of the isolate under investigation.

The conserved region 16S rRNA gene sequence of strain Naph2^T^, encompassing 1,318 bp, was sequenced and subsequently deposited in the GenBank database under the accession number ON062194. Taxonomic identification conducted via the EzTaxon server indicated that isolate Naph2^T^ belongs to the genus *Devosia*, with the highest 16S rRNA gene sequence similarity to *Devosia marina* L53-10-65^T^ (98.56%) and *Devosia subaequoris* HST3-14^T^ (98.33%), both of which fall below the 98.75% similarity threshold conventionally employed to define new species (Clarridge, 2004). Based on the observed differences in growth conditions and biochemical characteristics compared to related *Devosia* species, it was hypothesized that strain Naph2^T^ may represent a novel species within the genus *Devosia*. This hypothesis was further substantiated by phylogenetic analysis based on the 16S rRNA gene. The phylogenetic tree, constructed using the maximum likelihood method, revealed a significant genetic divergence between strain Naph2^T^ and its closest relative, *D. marina* L53-10-65^T^. The phylogenetic tree illustrated that strain Naph2^T^ formed a distinct and stable branch, and clusteredn with *D. marina* L53-10-65^T^ (NCBI accession number MN813764), *D. subaequoris* HST3-14^T^ (NCBI accession number AM293857), and *Devosia indica* (NCBI accession number MG852174), which shared a similarity below 98% to Naph2^T^ based on the conserved sequence alignment (Figure 1A). In conjunction with the physiological and biochemical characterization results (Table 1), strain Naph2^T^ demonstrated markedly distinct characteristics compared to its closest relatives, *D. marina* L53-10-65^T^ and *D. subaequoris* HST3-14^T^. These findings strongly support the classification of strain Naph2^T^ as a novel species of the genus *Devosia.*

### 3.3 OGRI supports the classification of Naph2^T^ as a novel species within the genus *Devosia*

Phylogenetic genomics based on 16S rRNA sequences provides crucial insights into the phylogenetic relationships of Naph2^T^, it does not adequately capture the comprehensive genomic similarity. Therefore, this study employed an OGRI, which includes dDDH, ANI, and AAI, to further assess genomic similarity. Accurate species identification is fundamental to microbial diversity analysis and functional comparison. As illustrated in Figure 1B, the digital dDDH analysis results for Naph2^T^ and other species within the genus *Devosia* were depicted in the upper right corner. The dDDH values between Naph2^T^ and its closest related species, *D. marina* L53-10-65^T^ and *D. subaequoris* HST3-14^T^, were 31.3 and 29.2%, respectively, which were significantly below the established species delineation threshold of 70% (Auch, n.d.). Similarly, the ANI analysis, presented in the lower left corner of Figure 1B, revealed ANI values between Naph2^T^ and *D. marina* L53-10-65^T^ was 88.2%, and with the *D. subaequoris* HST3-14^T^ was 86.6%, both of which were all below the 95% threshold for species separation (Jain et al., 2018). Additionally, the AAI values between Naph2^T^ and other type strains of the genus *Devosia* all fell below the 95% threshold commonly used for species classification (Jain et al., 2018) (Figure 1B). These findings indicate that Naph2^T^ is genetically distinct from other *Devosia* strains. Consequently, it has been proposed as a new taxonomic unit within the genus *Devosia*.

### 3.4 Degradation capabilities of Naph2^T^ for pyrene and benzo[a]pyrene

**Figure 2:**
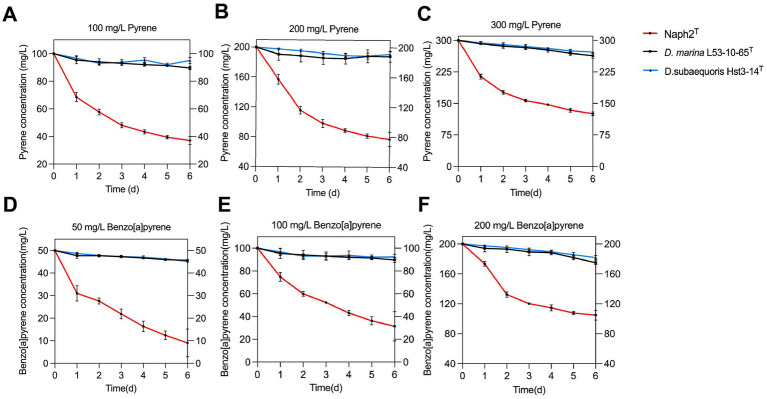
Biodegradation efficiency of the isolate Naph2^T^ and similar species was assessed when cultured with pyrene at concentrations of 100 mg/L **(A)**, 200 mg/L **(B)** and 200 mg/L **(C)**, and benzo[a]pyrene at concentrations of 50 mg/L **(D)**, 100 mg/L **(E)** and 200 mg/L **(F)**.

The degradation capacity of Naph2^T^ and related strains for pyrene and benzo[a]pyrene was evaluated by monitoring the reduction in concentration of these PAHs over time. Naph2^T^ cultures were incubated in MSM medium supplemented with 100 mg/L, 200 mg/L and 300 mg/L of pyrene, or 50 mg/L,100 mg/L and 200 mg/Lof benzo[a]pyrene, with the final results determined via HPLC. In the pyrene degradation experiments, degradation at 100,200 and 300 followed the same pattern. After two days of incubation, Naph2^T^ achieved degradation rates exceeding 40% for pyrene at concentrations of 100 mg/L, 200 mg/L, and 300 mg/L. Over the following four days, the degradation rate leveled off, and the degradation trend became more gradual. For 100 mg/L pyrene, Naph2^T^ achieved a degradation rate of 63% (Figure 2A), whereas for 200 mg/L, the degradation rate was 61.5% (Figure 2B) and slightly decreased to 58% for 300 mg/L pyrene (Figure 2C). Overall, the degradation proceeded rapidly during the initial 1–2 days, followed by a slower phase. At a benzo[a]pyrene concentration of 50 mg/L, Naph2^T^ showed a degradation rate of 38% on day 1, which remained stable from days 2 to 6 (Figure 2D). At 100 mg/L, the initial degradation rate was 26% on day 1, with minimal changes during the first three days and a slight increase thereafter (Figure 2E). At 200 mg/L, rapid degradation occurred during the first two days (34%), followed by only a 14% increase from days 2 to 6 (Figure 2F). Throughout the experiment, Naph2^T^ achieved degradation rates of 82, 69, and 48% for 50 mg/L, 100 mg/L, and 200 mg/L benzo[a]pyrene, respectively, in 30 mL of MSM. In contrast, the related strains *D. marina* L53-10-65^T^ and *D. subaequoris* HST3-14^T^ exhibited degradation rates below 10% for both pyrene and benzo[a]pyrene.

### 3.5 Genomic analysis of Naph2^T^ and the identification of functional genes involved in the degradation of PAHs

**Figure 3:**
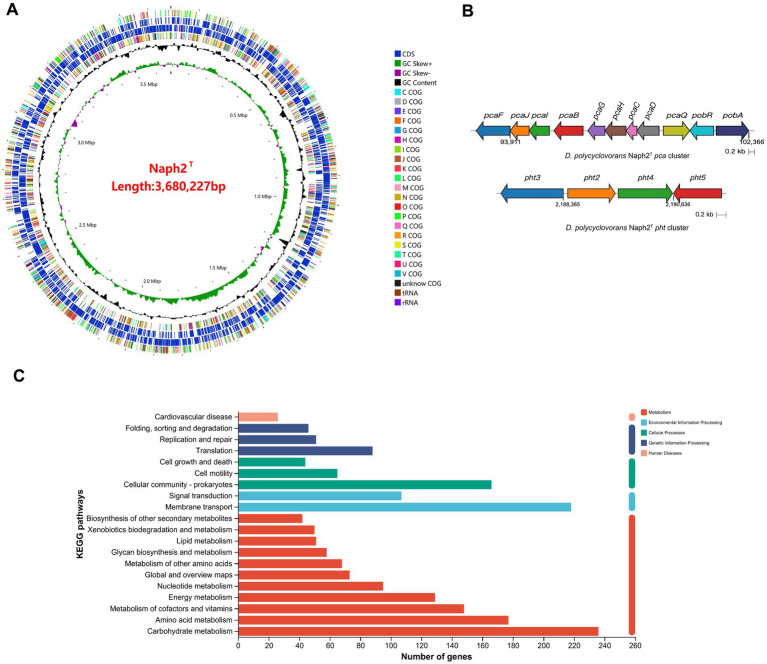
Genome analysis of Naph2^T^. **(A)** Circular map of Naph2^T^ genome. From inside to outside, the first circle represents the genome scale; Circle 2 is G + C skew; Circle 3 is the content of G + C; Circles 4 and 7 are clusters of orthologous groups; Circles 5 and 6 are locations of coding sequence, tRNA, and rRNA genes. **(B)** Prediction of functional gene clusters associated with PAHs biodegradation in Naph2^T^ genomes. **(C)** The KEGG annotations of the Naph2^T^ genome.

The whole genome of strain Naph2^T^ was found to comprise 4,445,453 bp with a G + C content of 61.50%, one main chromosome (3,394,779 bp with G + C content of 62.51%), and 3 plasmids. In addition to protein-coding sequences, the circular chromosome comprises 4 rRNA operons and 50 tRNA genes (Figure 3A). It also contains 6 gene islands and 2 CRISPR elements. The genomic differences between Naph2^T^ and typical strains that are evolutionarily related are presented in Table 2. By comparing the KEGG database as well as the NR database, 23 genes related to PAH degradation were annotated (Supplementary Table S1). The genome of Naph2^T^ contained the RHD, an aromatic ring hydroxylation dioxygenase gene encoding pyrene degradation, might play an critical function in the ring-opening process of LMW-PAHs and HMW-PAHs. In addition, the nagAa gene, located on both the genome and plasmid, encodes a ferredoxin reductase that transfers electrons from NAD(P)H to the dioxygenase system, enabling the incorporation of molecular oxygen into the aromatic ring—a key step in initiating aerobic PAH degradation. The *pht* gene cluster, located at 2187085–2191628 bp on the chromosome, is responsible for transformation of phthalic acid to 3,4-dihydroxybenzoic acid. The gene cluster consisted of 4 genes encoding phthalate 4,5-dioxygenase (*pht2*/*pht3*) (Arciero et al., n.d.), phthalate 4,5-cis-dihydrodiol dehydrogenase (*pht4*), and 4,5-dihydroxyphthalate decarboxylase (*pht5*), respectively (Figure 3B). Specifically, the *pca* gene cluster is responsible for breaking down 3,4-dihydroxybenzoic acid into smaller molecules that feed into the tricarboxylic acid (TCA) cycle (Overhage et al., 1999), while the *pob* gene cluster converts it into 4-hydroxybenzoic acid (DiMarco et al., 1993), allowing for further degradation (Figure 3B). Details of other functional genes involved in PAH degradation are provided in Supplementary Table S1.

The kegg annotation results show that: the majority of annotated genes were classified into metabolism-related pathways, including carbohydrate metabolism, amino acid metabolism, metabolism of cofactors and vitamins, and energy metabolism. Genes involved in xenobiotic biodegradation and metabolism, membrane transport, and signal transduction were also detected, indicating the strain’s potential for environmental adaptation and pollutant degradation (Figure 3C).

**Table 2:** Genomic information of Naph2^T^ and closely related strains.

Characteristic	1	2	3
Genebank accession number	GCA_042181575.1	GCA_009758415.1	GCA_009758415.1
Genome size (bp)	3,680,227	3,868,180	3,765,946
G + C content (%)	61.45	61.3	59.1
tRNA	50	49	47
rRNA	4	3	2
Plasmid	3	NA	NA
Gene island	6	1	0
CRISPERs	2	1	3
Genes	3,811	3,777	3,691

1, Naph2^T^; 2, *D. marina* L53-10-65^T^; 3, *D. subaequoris* HST3-14^T^. Genome size was calculated by Quast software, G + C content, plasmid, and genes number information was obtained from NCBI, tRNA was calculated by tRNAscan-SE, rRNA was calculated by Barrnap, Gene island was calculated by IslandPath-DIOMB, and CRISPERs were calculated by CRISPRCasFinder calculation, NA means unknown.

### 3.6 Comparative genomics analysis and genomic covariance analysis

**Figure 4:**
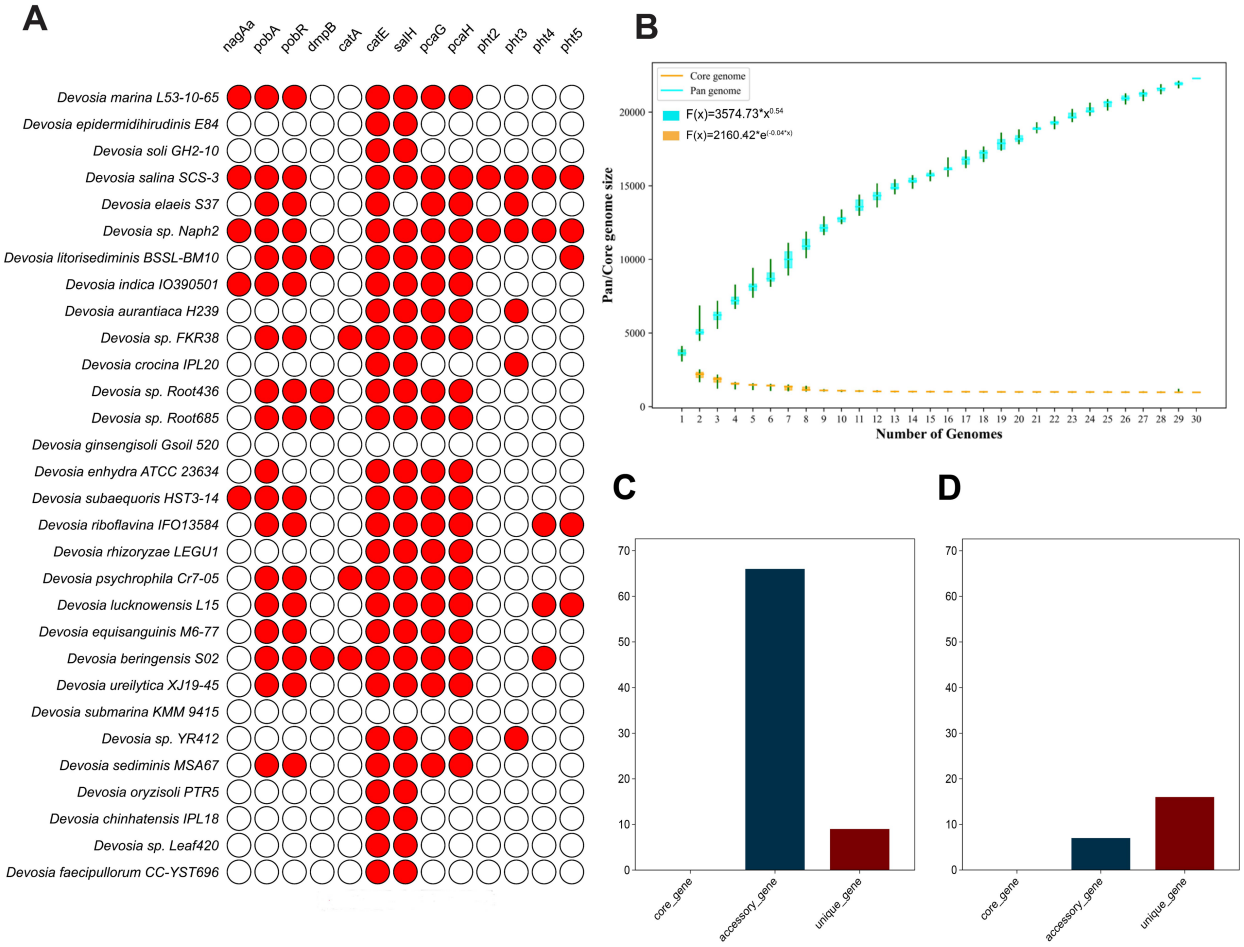
Comparative genomic analysis between Naph2^T^ with other closely related strains of the genus *Devosia*. **(A)** The distribution of key genes involved in PAHs degradation among *Devosia* strains. The circles in red indicates that the essential genes are present, and the white circles show that the genes are absent. **(B)** The boxplots of the pangenome (blue) and core genome (cyan) of the 30 analyzed genomes. **(C)** Distribution of genes related to PAHs degradation among core, accessory, and unique genes within the genus *Devosia*. **(D)** Distribution of the genes specifically in the Naph2^T^ genome.

Genes associated with PAH degradation in the genus *Devosia* are widely distributed across various strains. However, the abundance of these PAH degradation genes differs significantly among species (Figure 4A). In Naph2^T^, multiple PAH degradation genes and complete gene clusters—such as *pca*, *pht*, and *pob*—were identified. Furthermore, strains more closely related to Naph2^T^, including *D. marina* L53-10-65^T^ *and D. subaequoris* HST3-14^T^, were found to harbor a greater number of PAH degradation genes (Figure 4A).

Then we constructed a pangenome using 30 genomes of *Devosia* genuse, including Naph2^T^ and its closely related strains (Supplementary Table S2). Pangenome analysis revealed a total of 81,712 gene families, of which 983 (1.2%) were core genes, 68,118 (83.4%) were accessory genes, and 12,611 (15.4%) were unique genes (Supplementary Table S2). The number of accessory and unique genes differed substantially among the genomes (Supplementary Table S2). The genus *Devosia* displays an open pangenome, marked by an increasing number of accessory genes and a stable core genome as additional genomes are analyzed (Figure 4B). Interestingly, genes related to PAH degradation were primarily located in the variable and unique regions of both the *Devosia* pangenome and the Naph2^T^ genome. Interestingly, in the pangenome, the number of variable genes exceeded that of unique genes, while in Naph2^T^, unique genes were more numerous than variable genes. Specifically, Naph2^T^ possesses 983 core genes, 2,256 accessory genes, and 354 unique genes (Supplementary Table S2). Analyzing the annotations of core, essential, and unique genes revealed that PAH degradation genes in both *Devosia* and Naph2^T^ are primarily found among the variable and unique genes, with *Devosia* harboring a greater number of variable genes (Figure 4C) and Naph2^T^ containing more unique genes (Figure 4D).

**Figure 5:**
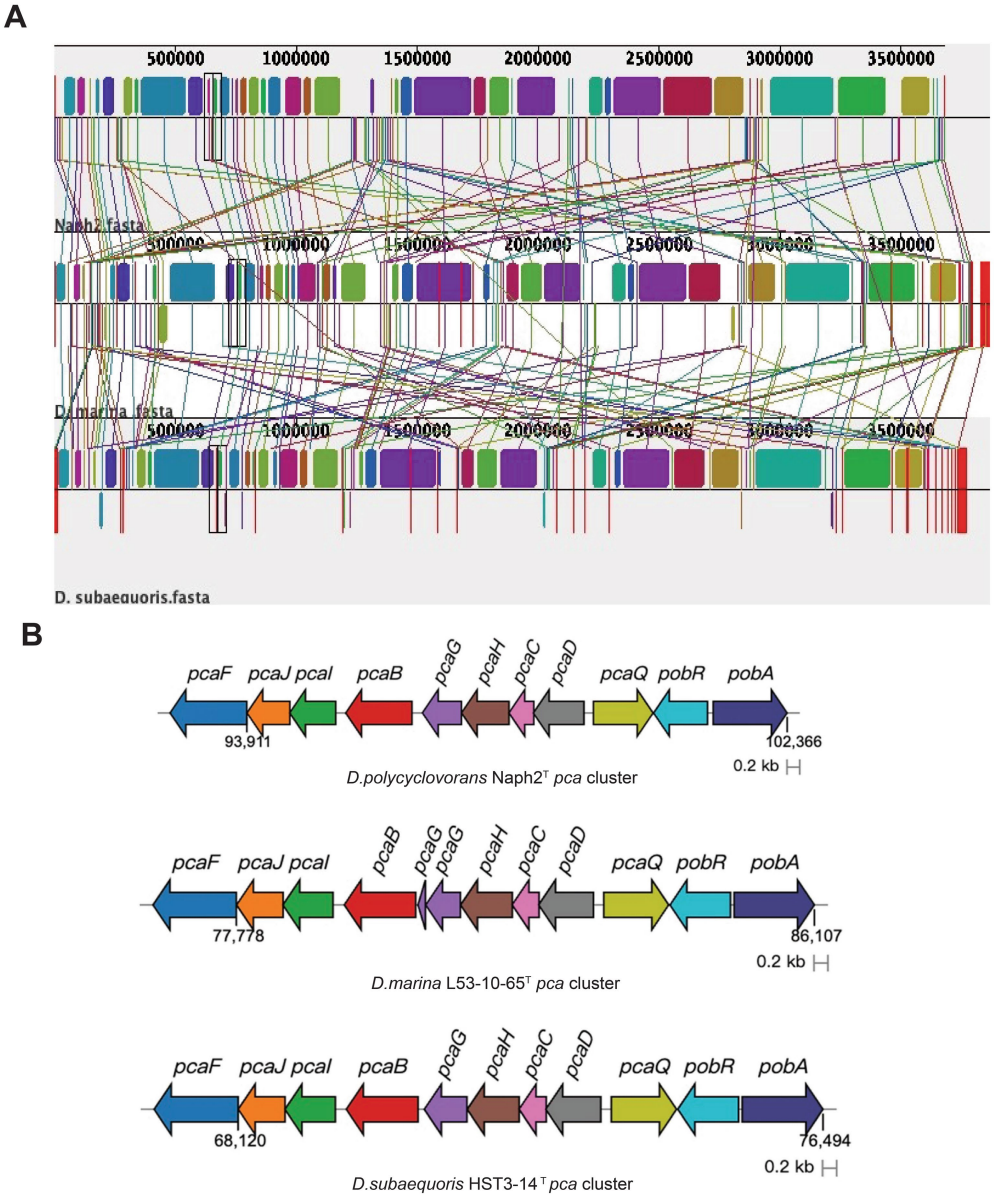
Genome synteny blocks and functional genes among species Naph2^T^, *D. marina* L53-10-65^T^, and *D. subaequoris* HST3-14^T^. **(A)** Genomic covariance analysis of Naph2^T^ and related strains *D. marina* L53-10-65^T^, *D. subaequoris* HST3-14^T^. Each horizontal line represents a genome, with color bars indicating distinct genes or genomic regions. Homologous genetic regions are denoted by similar colors. **(B)** Characterization of the *pca* gene cluster in strain Naph2^T^ and closely related strains, where different colors represent distinct genes.

In order to compare the genomic differences between Naph2^T^ and its closely related strains in more detail, we performed a genomic covariance analysis using Naph2^T^ as a reference strain. The results of the analysis showed that Naph2^T^ exhibited a high degree of homology with its close strains, *D. marina* L53-10-65^T^ and *D. subaequoris* HST3-14^T^, in most genomic regions (Figure 5A). For example, the *pca* gene cluster, which is involved in the degradation of 3,4-dihydroxybenzoic acid, is highly conserved in terms of gene arrangement, intergenic order and orientation (Figure 5B). These highly conserved genes may provide the strain with a strong metabolic capacity, e.g., in environmental remediation, especially in aromatic compound-contaminated environments. However, a *pht* gene cluster was present within the 2,100,000–2,200,000 bp region of the Naph2^T^ genome and absent in its closely related strains, and *phdK* and *phdE* genes involved in the upstream degradation of pyrene were missing in similar strains, as evidenced by the genomic covariance profile (Figure 5A). The absence of the *pht* gene cluster and other key degradation genes may explain the weaker PAH degradation ability in *D. marina* L53-10-65^T^ and *D. subaequoris* HST3-14^T^ (Figures 2A–D).

### 3.7 Transcriptional analysis to confirm the genes contributing to PAHs degradation

**Figure 6:**
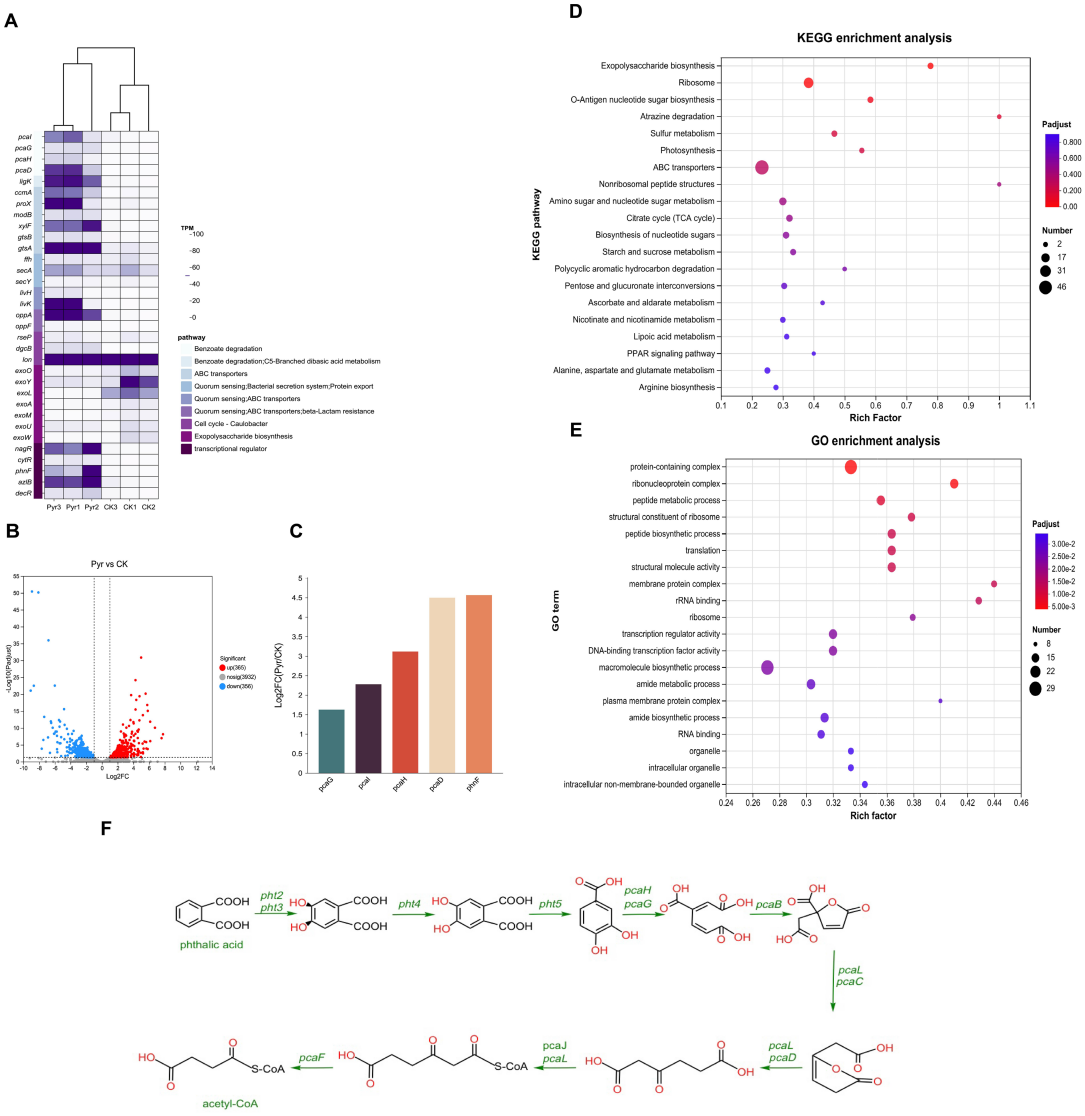
Transcriptome analysis of differentially expressed genes in culturing with pyrene as sole carbon source. **(A)** The heat map of difference expression genes in strain Naph2^T^ compared with the control. **(B)** Volcano plot of differentially expressed genes. The abscissa representing the log2 fold change and the ordinate representing the −log10 *p*-value. In this plot, the vertical line denotes the 2-fold change threshold, while the horizontal line indicates a *p*-value of 0.05. Each gene is represented by a point: red points (on the right) indicate up-regulated genes, blue points (on the left) indicate down-regulated genes, and gray points (at the bottom) indicate non-significant differentially expressed genes. **(C)** The expression levels of genes associated with pyrene degradation in strain Naph2^T^. **(D)** The enrichment analysis of KEGG pathways was conducted for differentially expressed genes induced by Naph2^T^. **(E)** The enrichment analysis of GO pathways was performed for these differentially expressed genes. **(F)** Downstream pathways involved in pyrene degradation by Naph2^T^.

To investigate potential gene clusters involved in the degradation of hypercyclic PAHs, we conducted a transcriptomic analysis of Naph2^T^ exposed to pyrene, using glycerol-treated cultures as the control group. The transcriptomic data indicated significant alterations in the key genes expression during pyrene degradation compared to the control group, which was grown in MSM supplemented with 1% glycerol. Applying a threshold of log_2_|fold change| > 1 and *p*-values < 0.05, we identified 365 up-regulated and 356 down-regulated genes relative to the control group (Figure 6B). Among the 365 significantly up-regulated genes, we further screened out 33 genes with large expression up-regulation. KEGG annotation analysis revealed that these genes were mainly involved in several important metabolic pathways and biological processes, including the benzoate degradation, ABC transporters, quorum sensing, exopolysaccharide biosynthesis and transcriptional regulator (Figure 6A). KEGG pathway enrichment analysis revealed that the differentially expressed genes were predominantly associated with pathways involved in arginine biosynthesis, alanine, aspartate and glutamate metabolism, and linoleic acid metabolism (Figure 6D). The metabolism of some of these amino acids provides important energy support for Naph2^T^, while the metabolism of fatty acids further enhances its ability to degrade organic pollutants by regulating the fluidity and stability of cell membranes. Additionally, GO enrichment analysis indicated that these genes were primarily linked to amide biosynthetic process and amide metabolic process (Figure 6E). Synthesis and metabolism of amide provide essential biomolecules for Naph2^T^ to support its key functions of energy supply, enzyme synthesis, and cellular repair during pyrene degradation.

The genes *pcaG* and *pcaH*, encoding 3,4-dihydroxybenzoic acid dioxygenase, which are responsible for the degradation of the intermediate metabolite 3,4-dihydroxybenzoic acid (Figure 6F), were highly expressed in response to induction of pyrene or intermediate metabolites (Figure 6C). Also, we identified 56 genes encoding hypothetical protein that showed a significant up-regulation trend, which may play an important role in pyrene degradation. However, we did not identify any genes that were highly similar to the reported pyrene dioxygenase genes, suggesting that new pyrene degradation genes with unknown functions may exist in these up-regulated gene clusters.

## 4 Discussion

The *Devosia* genus, belonging to the *Devosiaceae* family within the class *Alphaproteobacteria*, comprises 39 type species with formally recognized names.^7^ These bacteria are ubiquitously distributed across diverse global habitats, encompassing soil, animals, plants, sediments, wastewater, and are well known for their dominance in soil habitats contaminated with various toxins (Talwar et al., 2020). Additionally, *Devosia* spp. have been identified as prominent endophytes in the plants, such as Chinese silvergrass (*Miscanthus sinensis*) (Li et al., 2022) and rice (Jha et al., 2020), indicating their potential role in promoting plant growth and development. Of particular interest are *Devosia limi* D-8^T^ and *Devosia salina* SCS-3^T^, which have demonstrated efficacy in degrading the highly toxic mycotoxin deoxynivalenol (Vanparys et al., 2005; Pang et al., 2022). Despite these findings, research on the degradation of complex compounds and environmental pollutants by *Devosia* spp., as well as their broader ecological roles, remains insufficient. This study seeks to exploit deep-sea bacterial resources and augment the microbial degradation strains base for PAHs. In this study, we used a PAH as the sole carbon source for enrichment cultures and successfully isolated and identified a PAH-degrading strain, designated as Naph2^T^, from sediments collected in the Kermadec Trench. Phylogenetic analysis based on 16 s rRNA gene sequencing indicates that Naph2^T^ is affiliated with the genus *Devosia* (Figure 1). Whole-genome OGRI calculations further support that Naph2^T^ represents a novel species in the genus *Devosia*. Unlike previously characterized species of *Devosia*, Naph2^T^ exhibited a capacity to degrade HMW-PAHs, including pyrene and benzo[a]pyrene. In comparison to *Klebsiella michiganensis* EF4, a previously characterized PAH-degrading bacterium (Lou et al., 2023), Naph2^T^ completely degraded pyrene within six days at a concentration of 100 mg/L (Figure 2), while EF4 achieved only a 25% degradation rate. Additionally, Naph2^T^ exhibited an advantage over the marine PAH-degrading bacterium *Achromobacter* sp. HZ0, which took 30 days to degrade 60% of 100 mg/L pyrene (Deng et al., 2014). Fully elucidating the metabolic pathways involved in PAH degradation by strain Naph2^T^ is crucial for its potential use in the bioremediation of PAH-contaminated sites.

Genomic covariance analysis revealed that the *pca* gene cluster is highly conserved within Naph2^T^ and other phylogenetically related. This observation suggests that the stability and efficacy of the degradation pathway may have been maintained through strong selective pressure on the essential genetic components involved in PAHs degradation during evolution. However, the genome as a whole exhibits extensive gene rearrangements, gains, and losses, including the absence of the *pht* gene cluster in the PAH degradation pathway of evolutionarily related species. The significantly higher degradation of pyrene and benzo[a]pyrene by Naph2^T^ compared to its related strains, *D. marina* L53-10-65^T^ and *D. subaequoris* HST3-14^T^, was demonstrated. The RHD encoding genes, as annotated in the genome, did not exhibit significant variation in the transcriptome data, potentially due to the timing of sample collection. The degradation curves for pyrene indicated that the degradation capacity of Naph2^T^ for the substrate essentially reached its maximum after 4 days of fermentation at both treatment concentrations. This phenomenon may be linked to the decreased expression levels of upstream ring-opening genes. By aligning transcriptome sampling times with the PAH degradation profile of strain Naph2ᵀ, we observed that genes such as *pcaG*, *pcaH*, *pcaI*, and *pcaD*, which are associated with PAH degradation pathways, were transcribed at higher levels during the later stages of degradation, coinciding with the transformation of intermediate compounds. Furthermore, research has demonstrated the existence of two distinct downstream degradation pathways for pyrene, namely the phthalic acid and catechol pathways (Kim et al., 2005). In this study, transcriptome analysis revealed the differential expression of genes, suggesting that the downstream degradation pathway of strain Naph2ᵀ may proceed via phthalic acid (Figure 6). Specifically, the *pht* gene cluster facilitates the conversion of phthalic acid to 3,4-dihydroxybenzoic acid, which subsequently enters the tricarboxylic acid (TCA) cycle through the action of the *pca* gene cluster for further degradation into smaller molecules. The differentially expressed genes exhibited a pronounced up-regulation trend, particularly for those involved in ABC transporter proteins, quorum sensing pathways, exopolysaccharide biosynthesis, and transcriptional regulation (Figure 6), which are likely to play pivotal roles in pyrene catabolic processes. Consistent with previous studies, the up-regulation of genes encoding ABC transporter proteins may be linked to the translocation of substrates or the efflux of non-toxic small molecule metabolites (Ero et al., 2019). Furthermore, the activation of the quorum sensing system may enhance intercellular interactions, thereby augmenting the strain’s adaptability to complex environmental conditions, particularly in the presence of toxic PAHs (Mukherjee and Bassler, 2019). Additionally, the biosynthesis of polysaccharides may increase the strain’s resistance to environmental toxins, potentially by reinforcing the barrier function of the cell membrane or by secreting enzymes capable of degrading toxic substances (Pérez-Burgos et al., 2020). The upregulation of transcriptional regulators, conversely, plays a pivotal role in the coordinated regulation of intracellular gene expression, thereby ensuring the efficient functioning of the degradation pathway (Feng et al., 2016). Collectively, in the degradation process of pyrene, the differentially expressed genes were predominantly concentrated in the downstream metabolic pathways associated with the pollutant. Additionally, the transcription level alterations of the genes within other pathways contributed to the enhanced efficiency of pyrene degradation and the microbial adaptation to environments stressed by xenobiotics.

A pan-genomic analysis of the genus *Devosia* revealed that Naph2^T^ possesses a significantly greater number and diversity of genes contributing to PAHs degradation compared to other species, with these genes predominantly located in unique genes. The existence of unique genes is likely to be related to horizontal gene transfer, under PAH-enriched culture, bacteria may acquire PAHs degradation-related genes from other microorganisms through horizontal gene transfer, thus improving their competitiveness to survive in complex environments. Additionally, Naph2^T^ contains a copy of the *nagAa* gene on each chromosome and plasmid. Bacterial plasmids are typically primary vectors for horizontal gene transfer (Soucy et al., 2015). Through plasmid-mediated gene transfer, Naph2^T^ may have acquired the capacity to survive and maintain stability in complex environments, thereby enhancing its adaptability and degradation potential for pollutant remediation in challenging conditions (Soucy et al., 2015).

There are still several issues even if the current study offers a basic grasp of the Naph2^T^ degradation pathway. For instance, we can enhance the degradation of Naph2^T^ through adaptive evolution and overexpression of functional genes. In summary, this study introduces a novel species with potential for PAH pollution bioremediation and provides new insights into the ecological roles of the genus *Devosia*. Additionally, it highlights the species’ unique metabolic functions in deep-sea environments. The degradation capability of Naph2^T^ suggest that it could serve as a valuable candidate for bioremediation efforts targeting marine PAH contamination.

## 5 Conclusion

In this study, we isolated a strain of the genus *Devosia*, named *Devosia polycyclovorans* Naph2^T^ (po.ly.cy.clo.vo’rans. Gr. masc. Adj. polys, numerous; Gr. masc. n. kyklos, circle, ring; L. pres. part. vorans, devouring; N. L. part. Adj. polycyclovorans, degrading multiple-ring compounds), from the sediments of the Kermadec Trench using PAH-enriched cultures. Phylogenetic and OGRI analyses confirmed that Naph2^T^ is distinct from other closely related strains within the *Devosia* genus. Our results demonstrated that Naph2^T^ exhibits strong degradation abilities for high molecular weight PAHs, such as pyrene and benzo[a]pyrene, surpassing the degradation efficiencies of its closely related strains, *D. marina* L53-10-65^T^ and *D. subaequoris* HST3-14^T^. Genomic annotation of PAHS degradation genes, combined with dynamic transcriptomic expression analysis, revealed a significant increase in the expression of the *pca* gene cluster. Specifically, the expression levels of both small and large subunits encoding dioxygenases in this cluster were notably elevated. This suggests that the downstream pathway for pyrene degradation in Naph2^T^ involves the stepwise metabolism of 3,4-dihydroxybenzoic acid into small molecules that enter the TCA cycle. These findings highlight the robust potential of Naph2^T^ for pyrene degradation, indicating its capability to rapidly degrade environmental pyrene.

## Data Availability

The datasets presented in this study can be found in online repositories. The names of the repository/repositories and accession number(s) can be found in the article/Supplementary material.

## Footnotes

^1^ https://www.ezbiocloud.net/

^2^ https://www.ncbi.nlm.nih.gov

^3^ https://github.com/dparks1134/CompareM

^4^ https://www.chiplot.online

^5^ https://github.com/tseemann/barrnap

^6^ https://github.com/deweylab/RSEM

^7^ https://lpsn.dsmz.de/genus/devosia
