# Supplementary material for: Biodegradation of high-molecular-weight polycyclic aromatic hydrocarbons by a novel species of the genus Devosia isolated from the deep-sea region of the Kermadec Trench
Source: Front Microbiol. 2025 Jul 14;16:1584496. doi: 10.3389/fmicb.2025.1584496 (PMC12301985; doi:10.3389/fmicb.2025.1584496)
Supplement: Supplementary file 1 [file Supplementary_file_1.docx]

**Biodegradation of high-molecular-weight polycyclic aromatic hydrocarbons by a novel species of the genus *Devosia* isolated from the deep-sea region of the Kermadec Trench**

**Zefei Wang^a,1^, Shanshan Zhao^a,b,1^*****, Gen Chen^a^, Shiwei Sun^a^, Yue Liu^c^, Haixin Chen^c^, Liang Meng^c^, Zhuang Han^d^, Daoqiong Zheng^a^***

**^a^** Hainan Institute, Zhejiang University, Sanya 572025, China

**^b^** Donghai Laboratory, Zhoushan, Zhejiang 316021, China

**^c^** BGI Research, Sanya 572025, China

**^d^** Institute of Deep-sea Science and Engineering, 572000, China

* **Correspondence author**:

Email address: zhengdaoqiong@zju.edu.cn; zhaoshanshan5612@163.com

^1^ Zefei Wang and Shanshan Zhao contributed equally to this work.


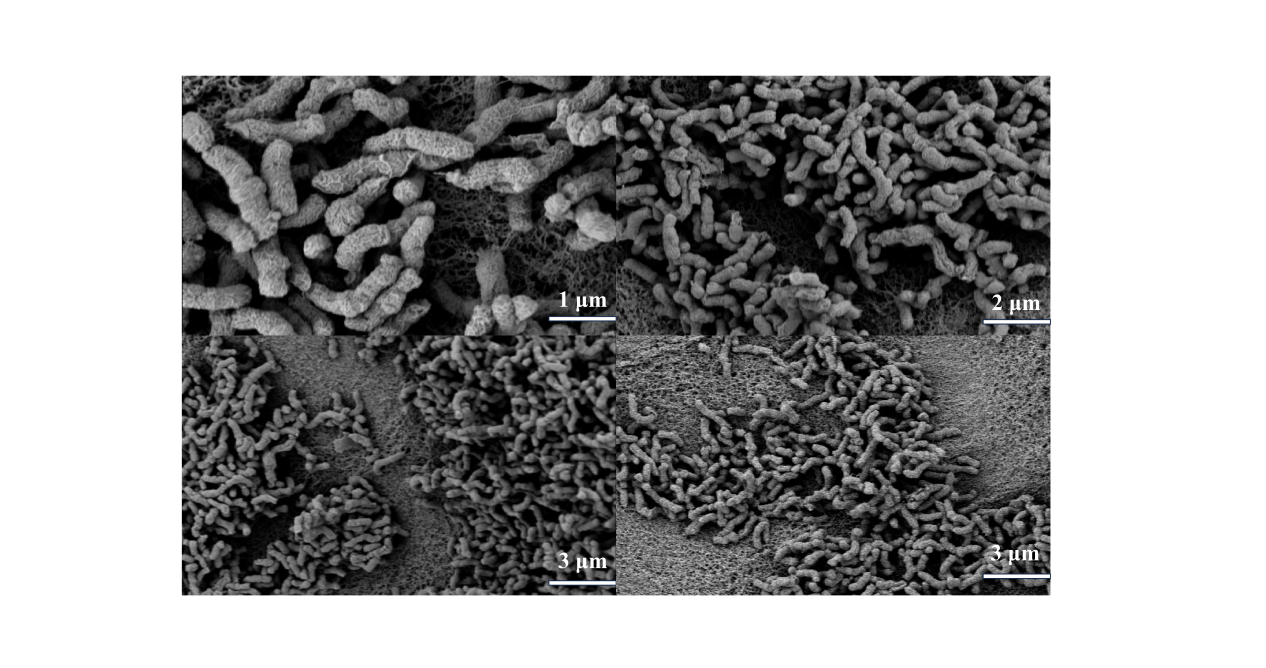


**Fig. S1**. Scanning electron micrograph of strain Naph2^T^ grown on 2216E agar.


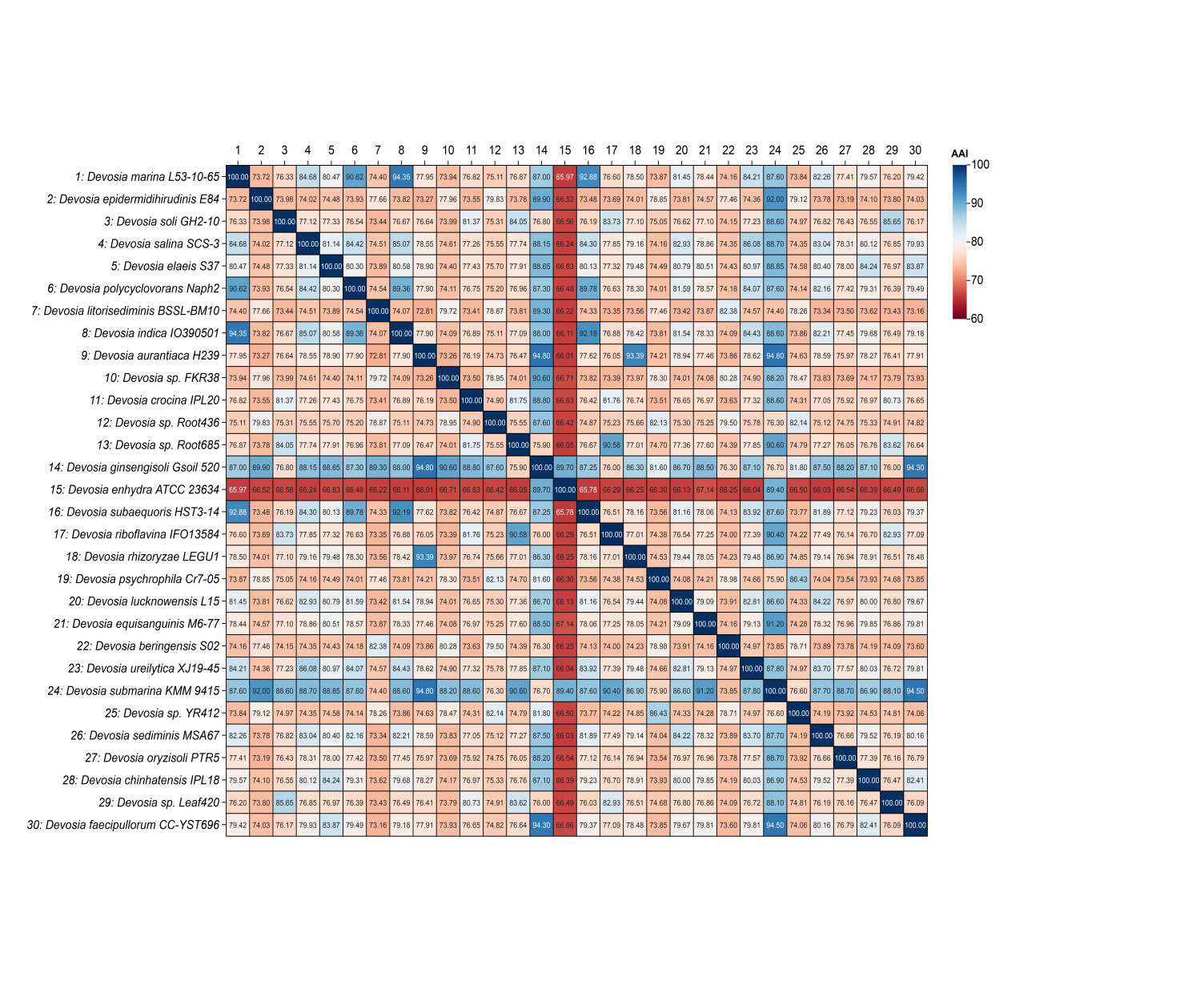


**Fig. S2.** The amino acid identity of strain Naph2^T^ with similar species within the *Devosia* genus.

**Table S1**

Putative genes associated with degradation of PAHs in Naph2^T^ genome

| **Gene id** | **Position** | **Genebank_number** | **Similarity(%)** | **Species** | **Gene name** | **Function description** |
| --- | --- | --- | --- | --- | --- | --- |
| 9 | 100275-101156 | AVF03224.1 | 82.97 | *Devosia sp*. I507 | pobR | transcriptional activator of pobA |
| 8 | 101209-102366 | MVT00077.1 | 93.25 | *Devosia marina* | pobA | 4-hydroxybenzoate 3-monooxygenase |
| 3 | 107420-108181 | BAA94705.1 | 34.2 | *Achromobacter xylosoxidans* | phdE | 2,3-dihydroxy-2,3-dihydrophenylpropionate |
| 2 | 1447493-1448386 | MVS99663.1 | 95.29 | *Devosia marina* | RHDs | aromatic ring-hydroxylating dioxygenase subunit alpha |
| 12 | 169442-170479 | QYO77286.1 | 98.12 | *Devosia salina* | pht4 | phthalate 4,5-cis-dihydrodiol dehydrogenase |
| 6 | 2045088-2046266 | MBB4051409.1 | 83.08 | *Devosia subaequoris* | salH | salicylate hydroxylase |
| 4 | 2174188-2175228 | BEG75972.1 | 54.2 | *Achromobacter xylosoxidans* | phdK | 2-formylbenzoate dehydrogenase |
| 10 | 2187085-2188365 | QYO77287.1 | 99.77 | *Devosia salina* | pht3 | phthalate 4,5-dioxygenase |
| 11 | 2188491-2189438 | QYO78935.1 | 97.38 | *Devosia salina* | pht2 | phthalate 4,5-dioxygenase reductase component |
| 13 | 2190636-2191628 | QYO77285.1 | 100 | *Devosia salina* | pht5 | 4,5-dihydroxyphthalate decarboxylase |
| 1 | 2822572-2823309 | AAD02138.1 | 28.9 | *Stutzerimonas stutzeri* | nahB | cis-dihydrodiol naphthalene dehydrogenase |
| 5 | 3332044-3333015 | MVT00493.1 | 97.52 | *Devosia marina* | NidD | alcohol dehydrogenase |
| 23 | 3678043-3679020 | MVT00138.1 | 97.22 | *Devosia marina* | nagAa | 2Fe-2S iron-sulfur cluster-binding protein |
| 7 | 54390-55313 | MVT00016.1 | 97.07 | *Devosia marina* | catE | catechol 2,3-dioxygenase |
| 14 | 92709-93911 | AVF03216.1 | 94.5 | *Devosia sp*. I507 | pcaF | acetyl-CoA acyltransferase |
| 15 | 93913-94584 | MBB4052940.1 | 97.76 | *Devosia subaequoris* | pcaJ | 3-oxoadipate CoA-transferase, beta subunit |
| 16 | 94581-95303 | AVF03218.1 | 97.5 | *Devosia sp*. I507 | pcaI | 3-oxoadipate CoA-transferase, alpha subunit |
| 17 | 95458-96495 | MBJ3786107.1 | 83.58 | *Devosia sediminis* | pcaB | 3-carboxy-cis,cis-muconate cycloisomerase |
| 18 | 96658-97269 | AVF03219.1 | 91.13 | *Devosia sp*. I507 | pcaG | protocatechuate 3,4-dioxygenase, alpha subunit |
| 19 | 97269-98015 | MBB4052944.1 | 98.79 | *Devosia subaequoris* | pcaH | protocatechuate 3,4-dioxygenase, beta subunit |
| 20 | 98012-98401 | AVF03221.1 | 92.19 | *Devosia sp*. I507 | pcaC | 4-carboxymuconolactone decarboxylase |
| 21 | 98394-99185 | MVT00074.1 | 82.13 | *Devosia marina* | pcaD | 3-oxoadipate enol-lactonase |
| 22 | 99333-100268 | MBB4052947.1 | 92.26 | *Devosia subaequoris* | pcaQ | pca operon transcriptional activator |

**Table S2**

Comparing genomic datasets and the number of individual gene sets.

| **Species** | **Strain name** | **Genebank number** | **Core_num** | **Accessory_num** | **Unique_num** |
| --- | --- | --- | --- | --- | --- |
| *Devosia aurantiaca* | H239 | GCA_011058215.1 | 983 | 1828 | 410 |
| *Devosia beringensis* | S02 | GCA_014926585.1 | 983 | 2461 | 305 |
| *Devosia chinhatensis* | IPL18 | GCA_000969445.1 | 983 | 1996 | 278 |
| *Devosia crocina* | IPL20 | GCA_900116545.1 | 983 | 2072 | 435 |
| *Devosia elaeis* | S37 | GCA_001650025.1 | 983 | 2207 | 454 |
| *Devosia enhydra* | ATCC 23634 | GCA_900119845.1 | 983 | 1418 | 1493 |
| *Devosia epidermidihirudinis* | E84 | GCA_000971295.1 | 983 | 1970 | 585 |
| *Devosia equisanguinis* | M6-77 | GCA_900631955.1 | 983 | 2388 | 447 |
| *Devosia faecipullorum* | CC-YST696 | GCA_015158295.1 | 983 | 1770 | 377 |
| *Devosia ginsengisoli* | Gsoil 520 | GCA_007859655.1 | 983 | 2497 | 629 |
| *Devosia indica* | IO390501 | GCA_003056405.1 | 983 | 2484 | 236 |
| *Devosia litorisediminis* | BSSL-BM10 | GCA_018334155.1 | 983 | 2199 | 312 |
| *Devosia lucknowensis* | L15 | GCA_900177655.1 | 983 | 2288 | 225 |
| *Devosia marina* | L53-10-65 | GCA_009758415.1 | 983 | 2324 | 268 |
| *Devosia oryzisoli* | PTR5 | GCA_014837245.1 | 983 | 2038 | 395 |
| *Devosia psychrophila* | Cr7-05 | GCA_000971275.1 | 983 | 2476 | 624 |
| *Devosia rhizoryzae* | LEGU1 | GCA_016698665.1 | 983 | 2158 | 214 |
| *Devosia riboflavina* | IFO13584 | GCA_000743575.1 | 983 | 3034 | 646 |
| *Devosia salina* | SCS-3 | GCA_019504385.1 | 983 | 2653 | 365 |
| *Devosia sediminis* | MSA67 | GCA_016411825.1 | 983 | 2474 | 457 |
| *Devosia soli* | GH2-10 | GCA_000970455.1 | 983 | 2550 | 329 |
| *Devosia sp. FKR38* | FKR38 | GCA_005222805.1 | 983 | 2151 | 415 |
| *Devosia sp. Leaf420* | Leaf420 | GCA_001425445.1 | 983 | 2562 | 331 |
| *Devosia sp. Root436* | Root436 | GCA_001426345.1 | 983 | 2296 | 235 |
| *Devosia sp. Root685* | Root685 | GCA_001427875.1 | 983 | 2265 | 446 |
| *Devosia sp. YR412* | YR412 | GCA_900110945.1 | 983 | 2713 | 249 |
| *Devosia polycyclovorans* | Naph2 | GCA_042181575.1 | 983 | 2256 | 354 |
| *Devosia subaequoris* | HST3-14 | GCA_024158985.1 | 983 | 2281 | 222 |
| *Devosia submarina* | KMM 9415 | GCA_003056345.1 | 983 | 2046 | 609 |
| *Devosia ureilytica* | XJ19-45 | GCA_024273205.1 | 983 | 2263 | 266 |
